# Procedural Sedation in Pediatric Gastroenterology: A Narrative Review from the Shared Perspective of Pediatric Gastroenterology and Anesthesiology

**DOI:** 10.3390/children13101294

**Published:** 2026-09-24

**Authors:** Sevim Cesur, Nihal Uyar Aksu, Ayşen Uncuoğlu, Tülay Çardaközü, Dilek İçli

**Affiliations:** 1 Department of Anesthesiology and Reanimation, Faculty of Medicine, Kocaeli University, 41001 Kocaeli, Türkiye; sevim.cesur@kocaeli.edu.tr (S.C.); tulay.hosten@kocaeli.edu.tr (T.Ç.); dozdamar@kocaeli.edu.tr (D.İ.); 2 Department of Pediatric Gastroenterology, Hepatology and Nutrition, Faculty of Medicine, Kocaeli University, 41001 Kocaeli, Türkiye; aysen.uncuoglu@kocaeli.edu.tr

**Keywords:** pediatric sedation, gastrointestinal endoscopy, propofol, ketamine, dexmedetomidine, remimazolam, capnography, high-flow nasal oxygen, risk stratification

## Abstract

**Highlights:**

**What are the main findings?**

- Propofol-, ketamine-, and dexmedetomidine-based regimens dominate pediatric gastrointestinal sedation; remimazolam and esketamine are emerging, but pediatric data remain limited.
- Capnography detects hypoventilation before desaturation, and high-flow nasal oxygen reduces hypoxemia in adults, but pediatric evidence remains limited and not yet statistically significant.

**What are the implications of the main findings?**

- Aim for the lightest effective sedation, matched to the procedure and a structured risk tier, with capnography-supported monitoring and consistent complication reporting.
- Because much of the pediatric evidence is extrapolated from adults, the efficacy of high-flow nasal oxygen and the role of remimazolam and esketamine require adequately powered pediatric trials before firm recommendations can be made.

**Abstract:**

**Background/Objectives:** Endoscopic and interventional procedures in pediatric gastroenterology are increasing in number and complexity, and most require sedation or, in selected cases, general anesthesia (GA); children face a higher risk of serious sedation-related adverse events than adults. Written jointly by pediatric gastroenterologists and anesthesiologists, this review provides a practical, evidence-referenced framework for procedural sedation across pediatric gastrointestinal procedures. **Methods:** We performed a narrative review of PubMed/MEDLINE (inception to 20 June 2026, the date of the final search), prioritizing society guidelines, systematic reviews, randomized controlled trials, and large registries on pediatric procedural sedation, sedative agents, capnography, and high-flow nasal oxygen (HFNO). **Results:** Sedation should be matched to the procedure and to a structured risk tier, targeting the lightest depth that allows safe completion. Propofol-, ketamine-, and dexmedetomidine-based regimens dominate; remimazolam and esketamine are emerging, but pediatric data are limited. Capnography detects hypoventilation before desaturation, and HFNO reduces hypoxemia in adults, whereas pediatric-specific evidence remains limited and the pediatric subgroup analysis did not reach statistical significance. Higher-risk groups require tailored planning, monitoring, and rescue readiness; most evidence is observational or extrapolated from adults. **Conclusions:** Safe pediatric gastrointestinal sedation combines structured risk assessment, rational drug combinations, capnography-supported monitoring, and consistent complication reporting. Who administers deep sedation, and whether general anesthesia is preferred, should depend on patient and procedural risk together with local regulations, institutional policy, training and credentialing, and rescue capability, within a shared gastroenterology-anesthesiology framework. Adequately powered pediatric trials of HFNO and of remimazolam/esketamine, and long-term neurodevelopmental follow-up, are priorities.

## 1. Introduction

Upper gastrointestinal endoscopy (esophagogastroduodenoscopy, EGD), colonoscopy, flexible sigmoidoscopy, capsule endoscopy, endoscopic retrograde cholangiopancreatography (ERCP), percutaneous endoscopic gastrostomy (PEG), and functional studies such as pH-impedance and manometry are all part of routine pediatric practice [1,2]. In children, procedural success depends on more than pain and anxiety control; it also depends on keeping the patient still, maintaining stable blood pressure and heart rate, lowering the risk of aspiration, and providing the endoscopist with a clear field to examine or treat [3,4].

Sedation management in children differs from that in adults. A relatively large tongue, a narrow subglottic space, a high oxygen demand of approximately 6–8 mL/kg/min, and a low functional residual capacity mean that apnea or hypoventilation can rapidly lead to hypoxemia [3,5]. Age-related shifts in volume of distribution, protein binding, and clearance make dosing harder to predict [5]. Comorbidities such as obstructive sleep apnea (OSA), reactive airway disease, congenital heart disease, and neuromuscular disorders increase the risk of complications and make a planned, multidisciplinary approach necessary [3,5,6].

The European Society for Paediatric Gastroenterology Hepatology and Nutrition (ESPGHAN) and the European Society of Gastrointestinal Endoscopy (ESGE) suggest performing pediatric endoscopy under general anesthesia or, only if general anesthesia is not available, under deep sedation in a carefully monitored environment [2,7]. The American Academy of Pediatrics (AAP) and the American Academy of Pediatric Dentistry (AAPD) set out a general, specialty-independent safety framework [5], and national societies continue to update pediatric sedation guidance [8].

Several contemporary reviews and guidelines already address pediatric endoscopic sedation [9,10,11]. The distinct contribution of this review is twofold: first, it is written jointly from the pediatric gastroenterology and anesthesiology viewpoints and makes explicit where these perspectives converge (patient safety, capnography, rescue capability) and where they legitimately differ (who administers deep sedation, and when GA is preferable); second, it integrates evidence published through 2026, including remimazolam and esketamine dosing and high-flow nasal oxygen (HFNO), which are absent from earlier syntheses. A recurring, practical decision is who should deliver sedation: anesthesiologist-delivered propofol-based deep sedation has become the most common regimen for pediatric endoscopy, whereas routine upper endoscopy and ileocolonoscopy generally do not require GA with endotracheal intubation, which may add risk and reduce efficiency [9]. The choice between deep sedation and general anesthesia, and who administers it, also depends on local regulations, institutional policy, practitioner training and credentialing, and demonstrated rescue capability, not only on patient- and procedure-related risk. The aim of this review is to provide a bedside-oriented, evidence-referenced framework for choosing the level of sedation, selecting agents, planning the airway and monitoring, and preventing and managing complications across specific patients and procedures.

## 2. Materials and Methods

This is a narrative review. We searched PubMed/MEDLINE for English-language articles from inception to June 2026 using combinations of the terms “pediatric/paediatric,” “procedural sedation,” “gastrointestinal endoscopy,” “esophagogastroduodenoscopy,” “colonoscopy,” “ERCP,” “propofol,” “ketamine,” “dexmedetomidine,” “remimazolam,” “capnography,” and “high-flow nasal oxygen.” Society guidelines (AAP/AAPD, ESPGHAN/ESGE, ASGE), systematic reviews, randomized controlled trials (RCTs), and large registry studies were prioritized, with the strongest and most recent pediatric evidence weighted most heavily; reference lists of retrieved articles were screened for additional sources. Titles and abstracts were screened for relevance to pediatric gastrointestinal procedural sedation; when several sources addressed the same point, the most recent and highest-level pediatric evidence was prioritized, and older primary studies were retained only when they remained the best available source. Because this is a non-systematic synthesis intended for clinical guidance rather than a quantitative review, no formal risk-of-bias assessment or meta-analysis was performed; the strength of the underlying evidence (guideline, RCT, registry/observational, or expert opinion) is indicated in the text where it affects interpretation. To help readers weigh the recommendations, key statements are qualified according to their strongest supporting evidence, namely pediatric guidelines or randomized trials, adult or observational data extrapolated to children, or expert consensus. As limitations inherent to this approach, the search was restricted to a single primary database (PubMed/MEDLINE) and to English-language publications, without Embase, the Cochrane Library, or a gray-literature search; selection and interpretation were therefore susceptible to selection and language bias, and the synthesis should be read as expert-guided rather than exhaustive.

## 3. Gastrointestinal Procedures and Their Sedation Needs

Pediatric gastroenterology procedures vary widely in duration, pain, airway involvement, and technical demand; therefore, the level of sedation and the choice of drugs must be matched to the procedure and the patient [1,2]. One feature that distinguishes pediatric from adult endoscopy is that biopsies are routinely taken from the esophagus, stomach, duodenum, and terminal ileum even when the mucosa looks normal, which lengthens the procedure and increases sedation requirements [1]. Pediatric and adult endoscopy differ in indications, equipment size, target sedation depth, and complication profiles [12].

### 3.1. Upper Gastrointestinal Endoscopy (EGD)

EGD is used to diagnose reflux and eosinophilic esophagitis, celiac disease, peptic disease, and inflammatory conditions, and for therapeutic work such as variceal ligation, foreign-body removal, stricture dilation, hemostasis, and the access step for PEG placement [1]. In young children, the gag reflex, anxiety, and the need to keep the patient still usually call for moderate-to-deep sedation, and deep sedation or GA is preferred for therapeutic work [3,4]. Because the endoscope occupies the mouth and limits airway maneuvers, oropharyngeal manipulation can trigger laryngospasm, and the team must be ready to rescue the airway [4].

### 3.2. Colonoscopy and Flexible Sigmoidoscopy

Colonoscopy is most often performed to assess inflammatory bowel disease and for polypectomy or to localize and treat lower gastrointestinal bleeding [1]. Visceral pain from distension and the length of the procedure often call for deep sedation, and GA may be considered for complex polypectomy or mucosal resection. Dehydration and electrolyte shifts after bowel preparation can cause hemodynamic swings, so fluids and electrolytes should be checked and corrected beforehand. Flexible sigmoidoscopy is shorter and less painful, and light-to-moderate sedation is usually sufficient.

### 3.3. ERCP

ERCP is used to diagnose and treat biliary and pancreatic disease. It is prolonged, is performed in the prone position, and involves extensive manipulation, which makes deep sedation difficult to maintain; GA is therefore often the safer choice [1]. In the prone position the airway is difficult to access, and the risks of aspiration and post-ERCP pancreatitis both warrant attention. Technical success and adverse-event rates in pediatric ERCP approach those in adults; a pediatric series summarized in the ASGE pediatric-practice guideline reported an overall post-ERCP pancreatitis rate of approximately 2.5% [1].

### 3.4. PEG, Capsule Endoscopy, Functional Studies, and Liver Biopsy

PEG placement requires analgesia and immobility and usually entails deep sedation; GA should be considered when aspiration risk is high or sepsis is suspected. Capsule endoscopy does not normally require sedation, although a young child who cannot swallow the capsule may need a short EGD under sedation for placement [1]. For pH-impedance and manometry, sedation alters motility and sphincter tone, so the goal is minimal sedation or none, with heavy reliance on behavioral preparation. Liver biopsy is usually performed with light-to-moderate sedation and adequate analgesia; coagulopathy, refractory ascites, or respiratory failure may necessitate GA and a secured airway. Foreign-body removal, esophageal button-battery removal, and ingestion of two or more magnets are emergencies, and the airway is frequently protected with endotracheal intubation under GA to guard against aspiration [1]. Typical sedation targets and points to watch are summarized in Table 1.

## 4. The Sedation Continuum and Pediatric Physiology

Sedation is a continuum extending from minimal sedation (anxiolysis) through moderate (conscious) sedation and deep sedation to GA [5,13]. The levels differ in the response to verbal or tactile stimulation, the integrity of airway reflexes, the adequacy of spontaneous ventilation, and cardiovascular stability (Table 2). Because the depth of sedation can increase without warning in children, the rescue principle is central: whoever administers sedation must be able to rescue a patient who has deepened one level beyond that intended, which requires the skills and equipment for airway management and ventilatory support [5,13,14]. In practice, minimal and moderate sedation are frequently and safely provided by appropriately trained non-anesthesiologists, including pediatric gastroenterologists, pediatricians, or specially trained nurses working under physician supervision, when competency-based training, capnography-supported monitoring, and demonstrated rescue capability are in place; deep sedation, and children with significant comorbidity or difficult-airway risk, more often warrant an anesthesiologist [5,11,13].

### Pediatric Physiology and Pharmacology

**Respiratory.** A high oxygen demand and low functional residual capacity mean that apnea or hypoventilation reduces saturation rapidly. The upper airway is small, and the soft tissues tend to collapse, particularly in OSA and obesity [3,5].

**Cardiovascular.** Cardiac output depends heavily on heart rate; bradycardia should therefore be treated as an early warning, whether it results from hypoxia, a vagal stimulus, or a drug.

**Pharmacokinetics.** A high total body water content can increase the volume of distribution of water-soluble drugs; hepatic enzyme maturation is age-dependent and accounts for some of the variability in propofol clearance [5]. In infants, lower protein binding leaves a larger free-drug fraction.

**Neurodevelopmental considerations.** Concerns about the effect of sedative and anesthetic exposure on the developing brain arise mainly from animal studies, whereas human data are reassuring for single, short exposures. In the randomized GAS trial, infants who received up to 60 min of anesthesia showed no difference in neurodevelopmental outcome at 2 years of age [15], a finding subsequently confirmed at 5 years of age [16]. The 2016 U.S. Food and Drug Administration (FDA) warning noted a possible risk with prolonged (>3 h) or repeated exposures in children under 3 years, while advising against delaying medically necessary procedures [17]. In practice, the priorities are to confirm that the procedure is needed, to minimize exposure time and the number of repeats, to maintain physiological stability, and to give families a balanced account of what is and is not known.

## 5. Procedure-Specific Sedation and Anesthesia

The sedation plan is more than the choice of drug: positioning, the airway plan, the analgesic component, and the flow of the case must all fit the procedure.

- **EGD.** The left-lateral position, adequate depth, a bite block, and control of secretions all help, and secretion control is one reason some teams favor ketamine or dexmedetomidine. A short diagnostic EGD is usually manageable under moderate sedation, whereas therapeutic work requires an analgesic component [3,4].
- **Colonoscopy.** Visceral pain from distension increases the need for analgesia. Adding a low-dose opioid or ketamine instead of using propofol alone can lower the total propofol dose (a balanced approach) but requires closer monitoring of ventilation [10,13].
- **ERCP.** Length, complexity, the prone position, and pancreatobiliary manipulation favor deep sedation or GA. If deep sedation is chosen, capnography and a written rescue-airway plan are essential, and supplemental oxygen is provided, with high-flow nasal oxygen considered in selected high-risk patients; dexmedetomidine–propofol or propofol–ketamine combinations balance stability against analgesia [1].
- **PEG placement.** This involves pain, aspiration risk, and gastric distension. It requires adequate analgesia (ketamine or an opioid), confirmation of fasting, and consideration of antibiotic prophylaxis [1].
- **Foreign-body or caustic ingestion (emergency).** When fasting is inadequate the aspiration risk rises; for button batteries and magnets the procedure is performed without delay, usually under GA with endotracheal intubation to protect the airway [1].
- **Functional studies.** Sedatives alter motility, so the target is minimal anxiolysis or no sedation, supported by behavioral preparation.

## 6. Pharmacologic Agents and Their Properties

No single agent is ideal; the aim is to balance analgesia, amnesia, and anxiolysis against respiratory and hemodynamic safety [3,18,19]. Titrating in small steps and combining agents provide synergy, while overlapping depressant effects (for example, propofol plus an opioid) should be minimized. Doses should be adjusted for age, weight (ideal or lean body weight in obesity), organ function, and concurrent medications. Onset, duration, dosing, advantages, cautions, and antagonists for the commonly used agents are summarized in Table 3, and rational combinations in Table 4.

### 6.1. Benzodiazepines

Midazolam acts on GABA-A to provide rapid anxiolysis and amnesia but no analgesia. It can be given intravenously (0.05–0.1 mg/kg; titrated, usual maximum total 0.1 mg/kg), orally (0.3–0.7 mg/kg; maximum 20 mg), or intranasally/buccally (0.2–0.3 mg/kg) [4,5]. Long the first-line agent in pediatric EGD, midazolam alone often provides inadequate sedation and is therefore combined with ketamine, propofol, or a narcotic such as meperidine or fentanyl [4,11]. Paradoxical agitation occurs in fewer than 2% of children, and reversibility with flumazenil is a real safety advantage [4]. Because the effect of midazolam outlasts that of flumazenil, patients should be observed for resedation. Remimazolam, an ultra-short-acting benzodiazepine rapidly metabolized by plasma esterases, is emerging: a recent systematic review of RCTs in pediatric upper gastrointestinal endoscopy found that it produced the fastest recovery among the evaluated regimens, although pediatric data remain limited and dosing/approval are institution-specific [20,21].

### 6.2. Opioids

Fentanyl (0.5–1 µg/kg IV) provides strong, predictable analgesia; a rapid bolus can cause chest-wall rigidity, and it also carries a risk of apnea and nausea. Sufentanil is more potent still (0.1–0.3 µg/kg), with a small injection volume but a narrow margin, and is generally reserved for anesthesiologist-led deep sedation with strict monitoring [18]. Alfentanil has a very fast onset and short action, and remifentanil is cleared rapidly by plasma esterases, which suits fine titration but demands careful capnography. Administering an intravenous opioid before the procedure, particularly within 30 min, is associated with a modest increase in oxygen desaturation, vomiting, and the need for positive-pressure ventilation [13]; naloxone should therefore be kept ready.

### 6.3. Hypnotics: Propofol and Etomidate

Propofol acts on GABA-A, has a fast onset (30–45 s) and a short duration that suit titration, and is antiemetic but not analgesic [18]. A typical bolus is 0.5–1 mg/kg (with 0.25–0.5 mg/kg top-ups), and for longer cases an infusion of 75–200 µg/kg/min is used. The main risks are dose- and rate-related hypotension, apnea, and injection pain (reduced by IV lidocaine 0.5 mg/kg). ESPGHAN describes propofol-based sedation as the safest and most practical way to reach deep sedation in pediatric endoscopy, with rare serious adverse events [2,4]. In large series delivered by specially trained pediatricians, the most common adverse events were desaturation (~0.4%) and laryngospasm (~0.2%) [4,20,22,23]. In pediatric endoscopic sedation, both propofol–ketamine and propofol–fentanyl combinations have proven effective and safe [24]. Etomidate (0.1–0.2 mg/kg) is hemodynamically neutral but causes myoclonus, nausea, and transient adrenal suppression; its use in pediatric endoscopy is limited [18].

### 6.4. The Dissociative Agent: Ketamine

Ketamine, an NMDA antagonist, provides dissociative sedation, strong analgesia, and amnesia (IV 0.5–1 mg/kg, with 0.25–0.5 mg/kg top-ups) [3,25,26]. It preserves spontaneous breathing and airway tone relatively well, causes bronchodilation, and its sympathomimetic effect lowers the risk of hypotension, an advantage in reactive airways or a fragile circulation [5,26]. In the Pediatric Sedation Research Consortium (PSRC) cohort of 22,645 children, serious adverse events with ketamine were rare, with an overall adverse-event rate of ~7.3% [25]; in a UK emergency-department series of 215 children, the most severe event reached only the “minor” category [27]. A systematic review of 67,871 children who received intravenous ketamine for emergency-department procedural sedation reported no deaths or permanent adverse outcomes, with sentinel airway or cardiovascular events in roughly 1 per 11,000 sedations, supporting the overall safety of ketamine in appropriately selected and monitored patients [28]. In pediatric GI endoscopy, laryngospasm has been reported in approximately 5% of ketamine sedations, with some earlier reports citing rates up to 10% [4,29]; all episodes were transient and resolved with airway positioning and supplemental oxygen. This figure derives from small upper-endoscopy series and is markedly higher than the ~0.3–0.4% laryngospasm rate seen in large mixed-procedure registries, most likely reflecting the added oropharyngeal stimulation of endoscopy rather than an intrinsic effect of ketamine. The mechanism is mechanical: pharyngeal and glottic stimulation by the endoscope, often compounded by secretions, provokes reflex laryngospasm, which is why a comparable excess is seen with other airway-manipulation procedures such as ear-nose-throat surgery (for example, adenotonsillectomy) but not with non-airway procedures. The practical implication is meticulous secretion control, an adequate plane of sedation, and airway vigilance rather than avoidance of ketamine. Hypersalivation (treated with glycopyrrolate 0.004–0.01 mg/kg), emergence reactions (a small dose of a benzodiazepine), and vomiting (prophylactic ondansetron) can all be managed [26]. Ketamine should be used cautiously in infants under 3 months and in active pulmonary disease or airway instability [4]. The effect of S-ketamine (esketamine) is dose-dependent: in an RCT synthesis, 0.3 mg/kg gave the shortest recovery, whereas higher doses (0.5–0.7 mg/kg) maintained heart rate better but caused more dizziness and visual disturbance [20].

### 6.5. The Alpha-2 Agonist: Dexmedetomidine

Dexmedetomidine is a selective central alpha-2 agonist that provides anxiolysis, opioid-sparing analgesia, and minimal respiratory depression [30,31]. The loading dose is 0.5–1 µg/kg over 10 min, followed by an infusion of 0.2–0.7 µg/kg/h. It can cause transient hypertension followed by bradycardia and hypotension; its slow onset and its tendency to be insufficient as a sole agent in long or stimulating procedures are its main limitations [31]. Because it spares respiration, it is useful in children with OSA and during sleep studies, and it can be combined with propofol or ketamine to reduce the total dose. In pediatric GI endoscopy specifically, a recent RCT found that nebulized dexmedetomidine improved gag-reflex suppression and sedation quality [32].

### 6.6. Combinations and Adjuncts

- **Ketofol (propofol + ketamine).** Ketamine’s sympathomimetic and respiratory-sparing effects offset propofol’s hypotensive and depressant effects, yielding steadier hemodynamics and often a lower total propofol dose; a common mix is 1:1 (mg:mg) titrated in small boluses [24]. In upper GI endoscopy, propofol–ketamine provided better hemodynamic stability than propofol–fentanyl [24].
- **Propofol + opioid (fentanyl).** Improves analgesia for painful manipulation such as dilation or polypectomy; capnography is mandatory because of additive respiratory depression [13].
- **Propofol + dexmedetomidine.** Lowers propofol use and may improve respiratory safety, at the cost of bradycardia and slower recovery.
- **Midazolam + narcotic (meperidine/fentanyl).** The most established conventional regimen; the availability of antidotes for both components (flumazenil and naloxone) is a clear safety advantage, but efficacy is often suboptimal and additional restraint or deepening may be needed [4].
- **Adjuncts.** IV lidocaine (0.5–1 mg/kg) eases propofol injection pain; ondansetron reduces nausea and vomiting with polypectomy or opioid use; anticholinergics control secretions.

Antidotes and rescue drugs—naloxone 0.01 mg/kg IV (repeat or infuse as needed); flumazenil 0.01 mg/kg IV (maximum single dose 0.2 mg; caution with a seizure history); atropine (0.02 mg/kg) or glycopyrrolate for bradycardia or hypersalivation; and ephedrine, phenylephrine, or epinephrine for refractory hypotension—should always be immediately available [4,5].

Table 3 summarizes the pharmacology and dosing of the commonly used agents; doses are starting ranges to be adjusted for the patient’s age, risk tier, and local protocol. Rational combinations are summarized in Table 4.

**Table 3:** Sedative, analgesic, and adjunct agents: pharmacology and dosing.

Agent	Pharmacologic Class	IV Onset	Peak (min)	Duration (min)	Typical Dose (Bolus/Infusion)	Advantage	Limit/Caution	Antagonist?	Notes
Midazolam	Benzodiazepine (GABA-A modulator)	1–2 min	3–5	20–40	0.05–0.1 mg/kg IV (max total 0.1)	Anxiolysis, amnesia	No analgesia; paradoxical agitation	Flumazenil	Core premedication for minimal/moderate sedation
Propofol	Alkylphenol IV hypnotic (GABA-A)	30–45 s	1–2	5–10 (bolus); longer on infusion	0.5–1 mg/kg boluses; inf 75–200 µg/kg/min	Rapid onset and recovery	Apnea, hypotension, injection pain	No (specific)	Not analgesic; add analgesia for painful work
Ketamine	Dissociative anesthetic (NMDA antag.)	30–60 s	1–2	10–20	0.5–1 mg/kg; top-up 0.25–0.5 mg/kg	Hemodynamic stability, strong analgesia, preserved reflexes	Hypersalivation, emergence, nausea	No (specific)	Antisialogogue + calm environment reduce emergence
Esketamine	NMDA antagonist (S-ketamine)	30–60 s	1–2	10–20	0.25–0.5 mg/kg (more potent)	Similar analgesia at lower dose	Hypersalivation, cost, emergence	None direct	Dose ~30–50% lower than ketamine
Dexmedetomidine	Selective alpha-2 agonist	5–10 min (load)	10–15	Infusion-dependent	Load 0.5–1 µg/kg (10 min); inf 0.2–0.7 µg/kg/h	Minimal respiratory depression, cooperative	Bradycardia, hypotension, slow onset	Atipamezole (not routine)	Slow titration; reversal not routine
Fentanyl	Phenylpiperidine µ-opioid	1–2 min	3–5	20–40	0.5–1 µg/kg; top-up 0.25–0.5 µg/kg	Strong, short analgesia	Resp. depression, chest rigidity	Naloxone	Small fractional boluses; capnography required
Sufentanil	Super-potent µ-opioid	1–2 min	3–5	30–50	0.1–0.2 µg/kg; top-up 0.1 µg/kg	Very potent, small volume	Dosing-error risk, resp. depression	Naloxone	Narrow window; pump/standard protocol
Etomidate	Imidazole IV hypnotic	30–60 s	1	5–15	0.1–0.2 mg/kg IV	Hemodynamically neutral	Myoclonus, nausea, adrenal suppression	No (specific)	Limited use in pediatric endoscopy
Lidocaine (IV adjunct)	Amide local anesthetic (Na+ block)	30–60 s	1–2	5–10	0.5–1.5 mg/kg (before propofol)	Lowers propofol need/injection pain	Toxicity at high dose	None (lipid rescue)	Watch cumulative dose (3–5 mg/kg)
Remimazolam	Ultra-short benzodiazepine (esterase)	1–2 min	3–5	Short (rapid offset)	0.05–0.1 mg/kg or weight-based infusion	Fast titration and recovery	Limited pediatric data, cost	Flumazenil	Approval/dosing institution-specific

Dose sources (mapped to the in-text references that report each dose): midazolam [4,5]; propofol [18] (safety data [2,4,22,23]); ketamine [3,25]; esketamine [20]; dexmedetomidine [30,31]; fentanyl [18] (peri-procedure timing risk [13]); sufentanil [18]; etomidate [18]; remimazolam [20,21]; antidote and rescue doses (flumazenil, naloxone, atropine, glycopyrrolate) [4,5]. The IV-lidocaine adjunct dose is a conventional range not tied to a single cited trial. All values are starting ranges to be individualized for age, weight, risk tier, and local protocol. Agent doses are supported by the cited pediatric procedural-sedation sources; remimazolam and esketamine pediatric doses are extrapolated from limited data and should be regarded as provisional.

**Table 4:** Selected combination regimens: goal, dosing, and monitoring.

Combination	Goal	Example Starting Doses	Expected Benefit	Specific Risk/Monitoring Focus
Propofol + ketamine	Hemodynamic and respiratory balance	Propofol 0.5 mg/kg + ketamine 0.5 mg/kg	Lower propofol need; stable BP	Emergence (ketamine); apnea (propofol)
Propofol + opioid	Add analgesia (e.g., polypectomy)	Propofol 0.5–1 mg/kg + fentanyl 0.5 µg/kg	Better pain control	Synergistic respiratory depression
Propofol + dexmedetomidine	Respiratory safety; less propofol	Dexmedetomidine load 0.5 µg/kg + propofol titration	More stable ventilation	Bradycardia; delayed recovery
Ketamine + dexmedetomidine	Counterbalanced hemodynamics	Ketamine 0.5 mg/kg + dexmedetomidine load 0.5 µg/kg	Less emergence; more stability	Bradycardia; salivation
Ketamine + midazolam	Anxiolysis + analgesia	Ketamine 0.5–1 mg/kg + midazolam 0.03 mg/kg	Less agitation/emergence	Respiratory depression (rare synergy)

Evidence basis differs across regimens: propofol–ketamine, propofol–opioid, and ketamine–midazolam are supported by pediatric endoscopy randomized trials, whereas propofol–dexmedetomidine and ketamine–dexmedetomidine rest on individual-agent pharmacology and expert consensus and should not be interpreted as equally evidence-based. Dose sources (mapped to the in-text references): propofol–ketamine (ketofol) and propofol–opioid regimens [24]; ketamine–midazolam [4,29]. Component doses are those given for each agent in Section 6 and Table 3. The propofol–dexmedetomidine and ketamine–dexmedetomidine starting doses are derived from each agent’s individual pharmacology [30,31] and expert consensus rather than a dedicated pediatric endoscopy trial; values are starting points requiring titration and capnography.

## 7. Risk Groups and Risk Stratification

A structured pre-procedure assessment reduces adverse events [1,5]. The American Society of Anesthesiologists (ASA) physical-status class summarizes overall perioperative risk and predicts adverse events in both adults and children, with ASA III and above carrying the highest risk [3,13]. Beyond ASA class, the airway should be assessed with the Mallampati score, mouth opening, jaw and mandibular anatomy, neck mobility, and tonsillar hypertrophy [3,13]. In a multicenter series of ~30,000 pediatric cases, the most common adverse events were desaturation, vomiting, excessive secretions, and unexpected apnea [19]; children with airway or cardiopulmonary problems (e.g., laryngomalacia, chromosomal anomalies, tetralogy of Fallot, bronchopulmonary dysplasia) therefore require extra care [3].

**Fasting (NPO).** Standard guidance is 2 h for clear liquids, 4 h for breast milk, and 6 h for formula or solids; the 2023 ASA Practice Guidelines for Preoperative Fasting reaffirm the 2 h clear-liquid window for pediatric patients undergoing elective procedures [1,5,33]. A recent noninferiority RCT further supports the safety of a 1–2 h clear-liquid window in children [34]; in patients at high aspiration risk (symptomatic reflux, gastroparesis, acute obstruction), GA with a secured airway may be preferred.

**Drug interactions.** Enzyme-inducing anticonvulsants can increase the sedative requirement, and chronic benzodiazepine or opioid use produces tolerance.

### Special Populations and Adjustments

- **Infants under 6 months.** Low reserve and a high metabolic rate cause rapid desaturation; use small incremental boluses, manage temperature, monitor with capnography, and extend post-procedure observation [3,5].
- **Obesity and OSA.** The upper airway collapses more easily and desaturation is more likely; a ramped or head-up position, an early airway adjunct, a minimal-opioid strategy, and dosing on ideal or adjusted weight all help. In OSA, dexmedetomidine- or ketamine-based protocols may be preferable.
- **Congenital heart disease.** In cyanotic lesions, a fall in systemic vascular resistance can increase right-to-left shunting; slow propofol titration, the hemodynamic margin of ketamine, and frequent noninvasive blood-pressure checks are all important.
- **Neurodevelopmental disorders.** Cooperation may be poor and paradoxical reactions may occur; a quiet room, parental presence, and ketamine or ketofol can help.
- **Hepatic or renal impairment.** Delayed elimination adds to the cumulative sedative load; widen dosing intervals and choose a short-acting agent where possible.
- **Recent upper respiratory infection.** Increased secretions and reflex reactivity raise the laryngospasm risk; weigh urgency against risk, and either postpone or plan a ketamine-led approach with gentle handling and secretion control [3]. In a recent systematic review, recent upper respiratory infection was one of three independent predictors of serious adverse events during pediatric ketamine sedation, together with age of 10 years or older and coadministered opioids [28].
- **Repeated sedation (IBD surveillance, serial endoscopy).** The long-term neurodevelopmental risk is unresolved; because a single short exposure appears reassuring [15] while cumulative exposure is best minimized [17], the sensible course is the lightest effective strategy, behavioral optimization, and combining elective procedures into a single setting when feasible.

## 8. Monitoring

The AAP guideline recommends continuous pulse oximetry and heart-rate monitoring at every level of sedation [5]. For moderate and deep sedation, noninvasive blood pressure, electrocardiography (ECG), and continuous assessment of ventilation should be added [5,13]. Vital signs and level of consciousness should be recorded at least before the procedure, after each drug dose, at least every 5 min during the procedure, during early recovery, and immediately before discharge [13].

**Capnography.** Because it detects hypoventilation and apnea before oxygen saturation falls, capnography is increasingly recommended for deep sedation [13]. In pediatric GI endoscopy and colonoscopy, adding capnography for non-intubated children reduced hypoxemia in randomized trials [1,35]. Adult endoscopy studies targeting deep sedation showed a similar reduction in transient hypoxemia, whereas its routine value under moderate sedation is less clear [13]. It is most useful in higher-risk phenotypes such as obesity, OSA, and prolonged procedures.

**Oxygenation.** Supplemental oxygen reduces the magnitude of desaturation during sedation; it should be considered for moderate sedation and administered for deep sedation [13]. High-flow nasal oxygen (HFNO) can extend the oxygen reserve during apnea or hypoventilation: in a meta-analysis of 19 RCTs (three in children), HFNO reduced hypoxemia during procedural sedation (risk ratio 0.37), lowered the need for minor airway maneuvers and procedure interruptions, and raised the minimum SpO_2_, with no significant effect on hypercarbia [36]. However, most of this evidence is from adults; in the pediatric subgroup (few trials) the reduction in hypoxemia did not reach statistical significance (risk ratio 0.33, wide confidence interval), with no significant difference between the adult and pediatric subgroups [36]. Pediatric-specific data are accruing: a randomized trial of transnasal humidified rapid-insufflation ventilatory exchange during sedated gastroscopy in children [37] and a pediatric meta-analysis of perioperative HFNO [38] both suggest benefit, and reviews of pediatric non-operating-room anesthesia (NORA) highlight the growing role of oxygen-delivery techniques and structured team workflows [39,40]. ECG is recommended for patients with cardiovascular disease or dysrhythmia and for prolonged procedures [13]. Evidence for EEG-derived depth monitors (BIS, entropy) in children is mixed, and they are not routinely required.

**Documentation and discharge.** Standardized discharge criteria should be used (e.g., AAP or ASA criteria, or an Aldrete score above 8). Cardiovascular function and airway patency should be stable, the patient should be easy to rouse with intact protective reflexes, and if a reversal agent (naloxone or flumazenil) was given, the patient should be observed long enough (up to 2 h) to detect resedation [4]. Infants and small children are at risk of airway obstruction if the head falls forward in a car seat, and families should receive written instructions about this [1,4].

### Oxygen-Delivery Techniques: A Comparison

These procedures are frequently performed in NORA settings, where registry data show that risk is broadly comparable to the operating room when patient assessment, room setup, monitoring, and simulation-based crisis response are standardized [40]. Oxygen delivery is one element the team must plan for; the principal options are compared in Table 5. Low-flow methods (nasal cannula, simple face mask, bite-block insufflation) are simple and widely available, whereas heated, humidified HFNO generates low-level positive airway pressure at high flow, washes out nasopharyngeal dead space, and improves oxygenation [36,37,38], but adds cost and requires equipment and training.

## 9. Tips and Tricks

### 9.1. Planning and Preparation

- Target the lightest level that allows safe completion; set the target from the length of the case, its pain intensity, and airway involvement.
- Use a structured pre-procedure checklist: (1) identity and indication; (2) fasting confirmation; (3) target sedation depth; (4) risk tier; (5) team roles; (6) airway plan A/B/C; (7) drugs and antidotes; (8) monitors on; (9) emergency equipment; (10) time-out sign-off.
- In anxious children, oral (0.5 mg/kg) or intranasal (0.2 mg/kg) midazolam premedication eases IV placement and separation from parents [1].
- Behavioral support (age-appropriate explanation, parental presence, virtual reality, play therapy, music) can lower the sedative requirement and improve cooperation.

### 9.2. During the Procedure

- Give small fractional boluses (e.g., propofol 0.5 mg/kg) and observe the clinical response for 60–90 s after each, rather than a start–stop approach, to avoid over-deepening in children.
- Balanced low-dose combinations (ketofol, or propofol plus a low-dose opioid) can improve hemodynamic stability and limit the dose of any single agent; in pediatric upper GI endoscopy, propofol–ketamine gave better hemodynamic stability than propofol–fentanyl [24].
- Preoxygenate (≥3–5 min of 100% O_2_ or 5–8 deep breaths); in higher-risk phenotypes keep a plan-B airway (nasopharyngeal airway, video laryngoscope) ready.
- In obesity or with lipophilic agents, dose by ideal or adjusted body weight and avoid overloading.
- For oral procedures, use nasal oxygen with integrated capnography (a second oxygen source) and position the bite block so it does not conflict with the scope.
- Ease propofol injection pain with IV lidocaine (0.5 mg/kg) or a larger vein; control secretions with an anticholinergic before or alongside ketamine.

### 9.3. Preventing and Managing Complications

- **Upper airway obstruction.** Jaw-thrust or mandibular advancement, sniffing position, oropharyngeal or nasopharyngeal airway, 100% O_2_, and positive-pressure ventilation if needed.
- **Hypoxemia thresholds.** SpO_2_ below 92% is an early warning; below 90%, pause and intervene; below 85% for ≥30 s or recurrent episodes warrants a protocol entry and event analysis [5,19].
- **Laryngospasm.** Stop the trigger, apply jaw-thrust and CPAP at 10–15 cm H_2_O with 100% O_2_; if unresolved, give a small propofol/deepening bolus, and if it persists, succinylcholine (0.5–1 mg/kg IV or 2–4 mg/kg IM) and secure the airway promptly [3,4].
- **Propofol-related hypotension.** Fluid bolus (5–10 mL/kg isotonic) and a slower rate; a vasopressor (ephedrine or phenylephrine) if it persists.
- **Dexmedetomidine-related bradycardia.** Atropine (0.02 mg/kg) if symptomatic, or reduce the infusion [31].
- **Ketamine emergence reaction or hypersalivation.** Reduce ambient stimulation and give a small dose of midazolam if needed; treat secretions with glycopyrrolate [26].
- **Rescue readiness.** In high-risk cases keep at least two staff competent in sedation, one with advanced airway experience; run annual crisis-resource-management simulation; and close the loop with post-event debriefs [3].

### 9.4. Quality and Safety Metrics

Track desaturation (SpO_2_ below 90% for >15 s), apnea, laryngospasm, the need for mask ventilation or an airway adjunct, airway conversion (LMA or ETT), procedure interruption, and rescue success. A standardized adverse-event classification (minor, moderate, major) lets centers compare results and improve over time [3,19]. Adverse events can occur regardless of the sedative, the combination, or the operator, which is why the quality of monitoring and the rigor of discharge criteria are non-negotiable [3,4].

## 10. Conclusions

Sedation for pediatric gastroenterology procedures should be patient-centered, individualized, and evidence-referenced. Careful pre-procedure assessment and risk stratification, a procedure-specific target, rational drug combinations, capnography-supported monitoring, and systematic reporting of complications together improve safety, efficiency, and the patient and family experience. Propofol-based protocols offer fast onset and recovery, while ketamine, dexmedetomidine, and opioid-sparing strategies can improve the respiratory and hemodynamic profile in specific subgroups; remimazolam and esketamine are promising but need more pediatric data. High-flow nasal oxygen likewise reduces hypoxemia in adults, but the pediatric benefit has not been confirmed in adequately powered trials and should be regarded as promising rather than established. The working relationship between the pediatric gastroenterologist and the anesthesiologist—especially in high-risk patients and in propofol- or ketamine-based deep sedation—is central. Priorities for future research include adequately powered pediatric RCTs of HFNO during endoscopic sedation, pediatric dosing and safety data for remimazolam and esketamine, validated pediatric risk-stratification and capnography protocols, and long-term neurodevelopmental follow-up after repeated sedation.

## Figures and Tables

**Table 1:** Typical sedation targets by procedure.

Procedure	Typical Sedation Target	Points to Watch
Diagnostic EGD	Moderate-to-deep sedation	Gag reflex; airway shared with scope; laryngospasm
Therapeutic EGD	Deep sedation/GA	Foreign body or battery (GA + intubation); bleeding; dilation pain
Colonoscopy	Deep sedation	Distension pain; dehydration and electrolytes after preparation
Sigmoidoscopy	Light-to-moderate sedation	Short; deepen if biopsy or analgesia is needed
ERCP	GA in most cases	Prone position; long duration; aspiration; post-ERCP pancreatitis
PEG	Deep sedation/GA	Aspiration; gastric distension; antibiotic prophylaxis
Capsule endoscopy	Usually none	Short EGD for placement if the child cannot swallow the capsule
pH-impedance/manometry	Minimal/none	Sedation alters the readings; use behavioral support

**Table 2:** Levels of sedation depth and their clinical features.

Level	Response to Stimulus	Airway/Ventilation	Cardiovascular
Minimal (anxiolysis)	Normal verbal response	Unaffected	Unaffected
Moderate (conscious)	Purposeful to verbal/tactile	Usually intact; mild hypoventilation risk	Usually stable
Deep	Purposeful to repeated/painful	May be depressed; intervention possible	Usually stable
General anesthesia	No response	Often needs intervention (LMA/ETT)	May be impaired

**Table 5:** Oxygen-delivery techniques during procedural sedation.

Technique	Mechanism/Typical Flow	Evidence in Sedation	Pediatric Notes/Limits
Low-flow nasal cannula	1–10 L/min; FiO_2_ depends on breathing	Standard; reduces desaturation magnitude [13]	Practical when the nose is free; baseline comparator
Simple face mask	≥5 L/min; higher FiO_2_ than cannula	Conventional method [13]	Competes with the oral scope in EGD
Bite-block/mouth-guard O_2_ insufflation	10–15 L/min; delivered around the scope	Conventional method in upper endoscopy [13]	Suits oral endoscopy; alternative to nasal route
High-flow nasal oxygen (HFNO)	Heated, humidified; up to ~70 L/min (adults), weight-based (children); low CPAP + dead-space washout	Reduces hypoxemia, airway maneuvers, interruptions; raises min SpO_2_; no hypercarbia effect [36,37,38]	Pediatric benefit suggested but subgroup not significant; cost/access limits [36,38]
Capnography-integrated nasal cannula	Nasal O_2_ + ETCO_2_ sampling	Detects hypoventilation before desaturation [13,35]	Suits oral (EGD) procedures

## Data Availability

No new data were created or analyzed in this study. Data sharing is not applicable to this article.

## Abbreviations

The following abbreviations are used in this manuscript:

AAP	American Academy of Pediatrics
AAPD	American Academy of Pediatric Dentistry
ASA	American Society of Anesthesiologists
EGD	Esophagogastroduodenoscopy
ERCP	Endoscopic retrograde cholangiopancreatography
ETT	Endotracheal tube
GA	General anesthesia
HFNO	High-flow nasal oxygen
LMA	Laryngeal mask airway
NORA	Non-operating-room anesthesia
NPO	Nil per os
OSA	Obstructive sleep apnea
PEG	Percutaneous endoscopic gastrostomy
PSRC	Pediatric Sedation Research Consortium
RCT	Randomized controlled trial
